# Sclerotherapy for hemorrhoidal disease: systematic review and meta-analysis

**DOI:** 10.1007/s10151-023-02908-w

**Published:** 2024-01-23

**Authors:** G. Gallo, A. Picciariello, C. Armellin, E. Lori, G. Tomasicchio, G. L. Di Tanna, G. A. Santoro, M. Alharbi, S. Sorrenti, U. Grossi

**Affiliations:** 1 Department of Surgery, Sapienza University of Rome, Rome, Italy; 2 Department of Experimental Medicine, University of Salento, Lecce, Italy; 3 Department of Surgery, Oncology and Gastroenterology - DISCOG, University of Padua, Padua, Italy; 4 Department of Business Economics, Health and Social Care, SUPSI – University of Applied Sciences and Arts of Southern Switzerland, Viganello-Lugano, Switzerland; 5 Imam Mohammad Ibn Saud Islamic University, Riyadh, Saudi Arabia

**Keywords:** Hemorrhoidal disease, Sclerotherapy, Polidocanol foam, Systematic review, Meta-analysis

## Abstract

**Background:**

This systematic review and meta-analysis aimed to evaluate the safety and efficacy of sclerotherapy methods for hemorrhoidal disease (HD) over the past 40 years.

**Methods:**

The review followed the 2020 Preferred Reporting Items for Systematic Reviews and Meta-analyses (PRISMA) guidelines. A comprehensive literature search was conducted, including studies reporting the use of sclerotherapy in patients with HD. Study eligibility criteria were defined, and data were extracted independently by the authors. Random-effects meta-analyses were performed to assess outcomes of interest.

**Results:**

Out of 1965 records identified, 44 studies met the inclusion criteria, involving 9729 patients. The majority of studies were conducted in Japan, followed by the UK, Italy, and Portugal. The median age of participants was 52 years, and the majority were male. The Goligher grade distribution indicated varying degrees of HD severity. Sclerotherapy was predominantly administered through anoscopy, with polidocanol being the most commonly used agent. The procedure was generally performed without pre-injection analgesia. The meta-analysis of 14 randomized controlled trials (RCTs) revealed that sclerotherapy was not inferior to control interventions in terms of success rate (risk ratio [RR] 1.00, 95% CI 0.71–1.41) and recurrence rate (RR 1.11, 95% CI 0.69–1.77), while resulting in fewer complications (RR 0.46, 95% CI 0.23–0.92).

**Conclusions:**

This systematic review highlights the safety and efficacy of sclerotherapy for HD, which yields similar success rates and fewer complications compared to other conservative or surgical approaches. Further research is warranted to optimize sclerotherapy techniques and evaluate long-term outcomes.

**Registration:**

PROSPERO 2023 CRD42023396910.

**Supplementary Information:**

The online version contains supplementary material available at 10.1007/s10151-023-02908-w.

## Introduction

Hemorrhoidal disease (HD) is one of the most common proctological diseases affecting the general population from mid-teens onward, with significant implications for national health services both in terms of surgeons’ workload and economic impact [1, 2].

Conventional surgical excisional or non-excisional treatments have shown high success rates but are also associated with increased pain and longer recovery periods compared to office-based procedures [3–5].

Sclerotherapy is a procedure indicated for grade I–II and grade III HD that is unresponsive to medical treatment. Additionally, it is effective in the symptomatic treatment of bleeding HD in elderly patients or those with severe comorbidities who are not suitable for traditional surgical interventions [6, 7]. While various methods and sclerosing preparations have been described in this context, most studies report the efficacy of liquid agents such as aluminum potassium sulfate and tannic acid [8], phenol in almond oil [9], and polidocanol [6]. Currently, polidocanol foam is one of the most widely used products, with apparently lower risk of complications compared to the liquid agents [10, 11]. It consists in the injection of sclerosing agents above the dentate line, directly into the internal hemorrhoidal plexus with consequent fibrosis and scarring of the hemorrhoids [5, 12].

The use of polidocanol in foam form has gained attention owing to its practical advantages, including potential cost-effectiveness, potential for fewer required office-based sessions, and possibly lower incidence of complications, which may contribute to good patient compliance [13, 14]. The foam formulation allows for a reduction in the injected dose of the sclerosing agent, potentially increasing the area of contact with the endothelium [15, 16].

The aim of this systematic review is to investigate methods of sclerotherapy for HD over the last 40 years and evaluate their safety and efficacy.

## Methods

The authors developed the protocol for review, detailing pre-specified methods of analysis and eligibility of the studies in line with the 2020 Preferred Reporting Items for Systematic Reviews and Meta-analyses (PRISMA) guidance [17]. The protocol was registered with PROSPERO on 5 March 2023 (CRD42023396910).

### Study characteristics

Search term definitions were inclusive, promoting a wide search of studies reporting the use of sclerotherapy in patients with HD. Studies were eligible regardless of whether they were retrospective or prospective in design, controlled or uncontrolled.

Studies were ineligible for inclusion if they described superseded series, out-of-scope procedures (e.g., sclerotherapy associated with other intervention(s) in the same patient [e.g., mucopexy or rubber band ligation]). Similarly, studies were excluded if outcomes could not be segregated for the index population (i.e., multiple or combined interventions for HD, where data were not stratified).

A minimum population sample of 15 adult subjects (index population) was imposed for eligibility. This pragmatic threshold excluded case reports and small case series that often reported on early experience with the techniques.

### Report characteristics

Any publication date was eligible from 1 January 1983 to the date of the final search performed on 1 December 2022. As a result of the large number of studies retrieved, it was decided to include only studies with full-text publications written in English. Only peer-reviewed publications reporting primary data were eligible. Thus reviews, editorials, and letters were excluded at the screening stage. Conference abstracts and proceedings were also excluded.

### Information sources and study selection

The authors performed a comprehensive search of the literature using Medline (PubMed), Web of Sciences, Scopus, and EMBASE and hand-searching using all common search terms encompassing sclerotherapy with synonymous variants (i.e., [hemorrhoids] AND [sclerotherapy or injection or foam or polidocanol or sclerofoam]). Reference lists of all full-texts were hand-selected for any additional studies.

### Data extraction

Screening was independently performed at the title and abstract levels by three coauthors (CA, EL, and GT), excluding studies not meeting eligibility criteria where these could be readily determined from the title/abstract alone. Full-text copies of all remaining studies were also obtained and assessed by the junior authors, who were un-blinded to the names of studies, authors, institutions, or publications. Disagreement regarding inclusion was resolved by consensus. Study characteristics and outcome data were extracted into a Microsoft Excel spreadsheet (XP professional edition; Microsoft Corp, Redmond, Washington, USA).

First author, publication year, country of origin, reason for exclusion, and type of study were extracted for each study, and the following data for each arm: Goligher grade, route of administration of sclerotherapy (i.e., anoscopic or endoscopic), type of sclerosing agent, type of needle, method of formation of the injected product, site(s) of injection, injected volume (total and per pile), patient position, anesthesia, study length (months), number of patients, number of male patients, mean or median age, mean injection time, intra- and postoperative complications, pain, success rate (overall and after the first injection), recurrence rate, follow-up (months), and scoring system(s).

We assessed the risk of bias in randomized controlled trials (RCTs) using the JBI Critical Appraisal Tool for Assessment of Risk of Bias [18], which provided a structured approach to evaluating study quality and potential sources of bias.

### Statistical analysis

A narrative synthesis of the studies was reported for the included studies. Meta-analyses were limited to RCTs based on the type of sclerotherapy for HD and for each outcome of interest (overall morbidity, postoperative pain, success, and recurrence rates). Acknowledging heterogeneity across studies we fitted random-effects models using the Sidik-Jonkman-Hartung-Knapp estimate of the heterogeneity. Statistical heterogeneity was assessed by formal test of homogeneity and evaluating the proportion of variability attributable to heterogeneity rather than sampling error (*I*^2^). Small study effects were assessed by evaluation of the funnel plot and via regression-based Egger test. Statistical analyses were performed using STATA V.17 (StataCorp, College Station, Texas, USA).

## Results

### Study selection

**Fig. 1:**
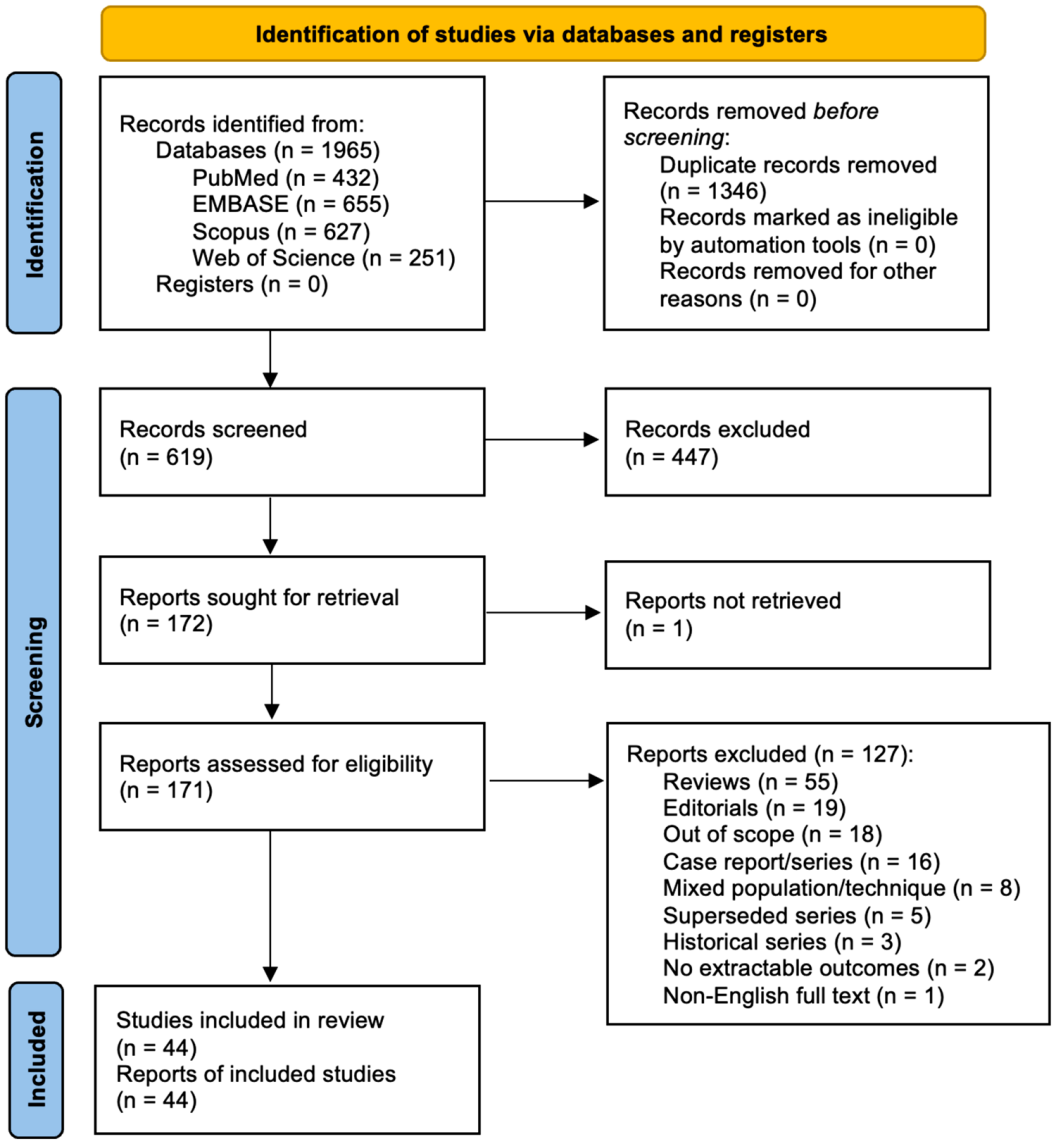
PRISMA diagram

After removal of duplicates from a total of 1965 identified records, 619 were screened. Among these, 447 (72.2%) were excluded (Fig. 1). One report could not be retrieved [19].

Overall, 44 studies published between 1985 and 2022 met the inclusion criteria and reported data on 9729 patients. These studies included 17 RCTs [3, 14, 20–34], 1 case–control study [35], and 26 cohort studies (Table 1). The majority of the studies were conducted in Japan (*n* = 9) [8, 35–42], followed by the UK (*n* = 6) [20–23, 43, 44], Italy (*n* = 6) [12, 45–49], and Portugal (*n* = 4) [3, 33, 50, 51]. Six (14%) of the studies were multicenter [14, 35, 41, 47, 51, 52].

**Table 1:** Characteristics of the included studies

First author, reference	Year	Country	Study type	No. patients	Recruitment time (months)	Index injected agent
Ambrose [20]	1985	UK	RCT	62	NR	5% phenol in oil
Khoury [21]	1985	UK	RCT	62	35	5% phenol in oil
Gartell [22]	1985	UK	RCT	109	72	5% phenol in oil
Senapati [23]	1988	UK	RCT	23	NR	5% phenol in oil
Mann [43]	1988	UK	PCS	100	8	5% phenol in oil
Jamjoom [58]	1991	Saudi Arabia	PCS	280	36	5% phenol in oil
Ponsky [54]	1991	USA	RCS	18	NR	23.4% saline
Varma [24]	1991	Hong Kong	RCT	28	NR	5% phenol in oil
Santos [44]	1993	UK	RCS	189	12	5% phenol in oil
Jaspersen [25]	1993	Germany	RCT	40	NR	5% phenol in oil
Kanellos [62]	2000	Greece	RCS	240	72	5% phenol in oil
Kanellos [26]	2003	Greece	RCT	80	48	5% phenol in oil
Khan [27]	2006	Pakistan	RCT	52	8	5% phenol in oil
Takano [35]	2006	Japan^a^	CC	80	36	OC-108
Benin [45]	2007	Italy	RCS	250	NR	STS foam
Yuksel [28]	2008	Turkey	RCT	62	24	3% polidocanol liquid
Hachiro [8]	2011	Japan	RCS	448	50	ALTA
Awad [29]	2012	Egypt	RCT	60	24	5% EO or N-BC
Moser [14]	2013	Germany^a^	RCT	66	25	3% polidocanol foam
Tokunaga [36]	2013	Japan	PCS	940	38	ALTA
An [52]	2014	China/Japan^a^	PCS	760	72	An’s Shaobei
Yano [38]	2014	Japan	PCS	57	60	ALTA
Zhang [55]	2015	China	PCS	30	NR	Polidocanol
Yano [37]	2015	Japan	RCS	55	12	5% phenol in oil
Tomiki [40]	2015	Japan	PCS	83	36	ALTA
Miyamoto [41]	2016	Japan^a^	RCS	169^b^	73	ALTA
Akindiose [59]	2016	Nigeria	PCS	40	18	5% phenol in oil
Shah [30]	2018	India	RCT	25	NR	3% polidocanol liquid
Tomiki [39]	2019	Japan	PCS	33	36	ALTA
Fernandes [50]	2019	Portugal	PCS	2000	68	2% polidocanol foam
Ronconi [46]	2019	Italy	RCS	615	132	3% polidocanol foam
Abiodun [31]	2020	Nigeria	RCT	30	12	50% dextrose water
Makanjuola [53]	2020	Nigeria	PCS	37	12	3% polidocanol liquid
Mishra [34]	2020	India	RCT	75	15	3% polidocanol liquid
Shafi [32]	2021	Pakistan	RCT	60	12	3% polidocanol liquid
Lobascio [12]	2021	Italy	RCS	66	17	3% polidocanol foam
Neves [33]	2022	Portugal	RCT	24	6	3% polidocanol foam
Gallo [47]	2022	Italy^a^	PCS	183	6	3% polidocanol foam
Xie [56]	2022	China	RCS	201	45	1% polidocanol liquid
Abe [42]	2022	Japan	RCS	1180	96	ALTA
Salgueiro [51]	2022	Portugal^a^	PCS	228	19	3% polidocanol foam
Salgueiro [3]	2022	Portugal	RCT	60	NR	3% polidocanol foam
Goglia [48]	2022	Italy	PCS	50	3	3% polidocanol foam
Lisi [49]	2022	Italy	PCS	19	14	3% polidocanol foam

*RCT* randomized controlled trial; *RCS* retrospective cohort study; *PCS* prospective cohort study; *CC* case–control study; *ALTA* aluminum potassium sulfate and tannic acid; *EO* ethanolamine oleate; *N-BC N*-butyl cyanoacrylate; *STS* sodium tetradecyl sulfate; *NR* not reported; An’s Shaobei is a Chinese herbal remedy containing extracts of herbs, i.e., citric acid, gallic acid, paeoniflorin; adjuvant material was aseptic sterile water for injection

^a^Multicenter

^b^Grade III

### Demographic and clinical characteristics

In 39 (89%) of the studies, the median age of the participants was 52 years (interquartile range 47–56). Thirty-eight (86%) studies reported the gender of the patients, comprising a total of 9170 (94.3%) subjects, of which 5535 (62.1%) were male. The median recruitment time was 25 months (range 3–132) in 36 (82%) of the studies.

Data on Goligher grade were available in 33 (75%) studies, including 7480 (77%) patients. Among them, 632 (8.4%) had grade I, 3259 (43.6%) had grade II, 3309 (44.2%) had grade III, and 280 (3.7%) had grade IV HD. Only 7 (16%) studies (all published between 2020 and 2022) incorporated validated scores for HD [3, 12, 33, 47, 48, 51, 53].

### Procedure

Only eight studies described endoscopic administration [29, 31, 39, 40, 46, 54–56], while anoscopy was the adopted route in the remaining studies. The most frequently injected agent was polidocanol (*n* = 17 [39%] studies) in different states (liquid or foam; Table 1) and at different concentrations (1–3%), followed by 5% phenol oil (*n* = 15 [34%] studies). The needle’s caliber ranged between 20 and 25 gauge in 14 (32%) studies. Tessari’s method [57] was used to prepare the foam of polidocanol in nine studies [3, 12, 14, 33, 46, 47, 49–51], and the Varixio system in one study [48].

The site of injection was submucosal intra-hemorrhoidal in 25 (57%) studies, at the anorectal junction in seven studies [3, 12, 24, 31, 41, 46, 58], around the three afferent branches of the superior rectal artery in one study [25]. In another study, each pile was injected once, in several directions, and at variable depths [50]. The median injected volume per pile and total volume were 3 ml (interquartile range 3–4.5) and 9 ml (9–15), respectively, for 5% phenol oil (*n* = 12 studies); 2 ml (1.5–2) and 5 ml (4.5–5.5) for polidocanol liquid (*n* = 5 studies); and 2 ml (2–2) and 6 ml (6–7) for polidocanol foam (*n* = 10 studies).

Patient positioning was described in 24 (54%) studies. The majority of the injections were performed in the Sims position (*n* = 20 studies), while a few authors described the jack-knife [37, 42] and the lithotomy position [34, 41] as alternatives.

The procedure was performed without the use of pre-injection analgesia in most studies. Some authors preferred local analgesia [31, 36, 39, 41, 59], while lumbar analgesia was limited to Japanese authors [8, 35, 37, 42].

The median procedural time was 8.5 min (6.5–12) in 11 studies. Pain (mostly mild) was the most frequent perioperative issue,[14, 27, 48, 59] and typically occurred in 8% (3–13%) of cases in 30 (68%) studies. Bleeding affected a mean of 3.4% of patients in 18 (41%) studies. Other minor complications were rarely reported, such as urinary retention, local edema, tenesmus/discomfort, pruritus, paraesthesia, and external thrombosis. The median follow-up was 12 months (3–12), with 11 (25%) studies reporting a follow-up exceeding 12 months. Overall, 87% (67–98%) of patients were satisfied with the treatment in 9 (20%) studies.

### Meta-analyses of RCTs

**Fig. 2:**
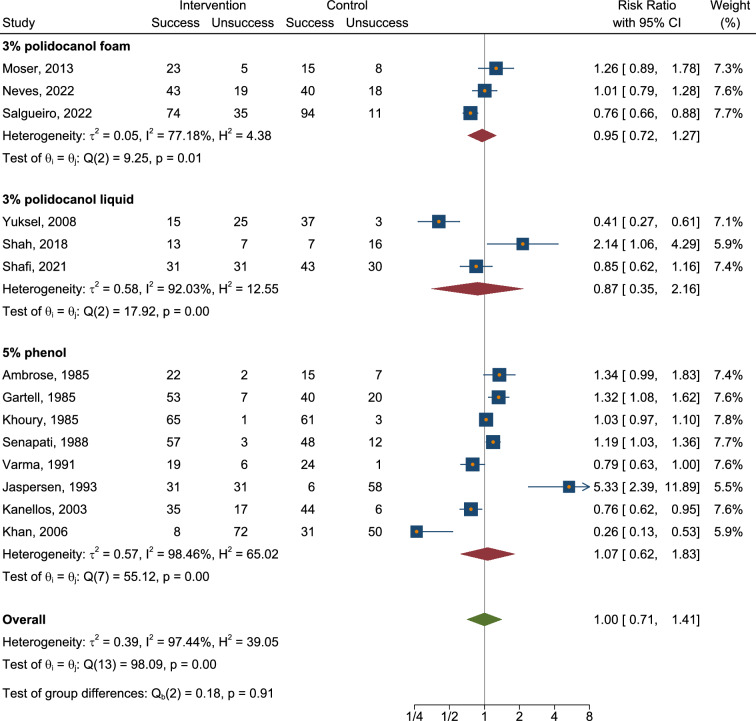
Forest plot showing rates of success

**Fig. 3:**
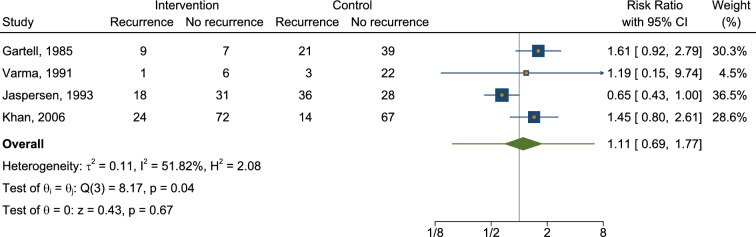
Forest plot showing rates of recurrence

**Fig. 4:**
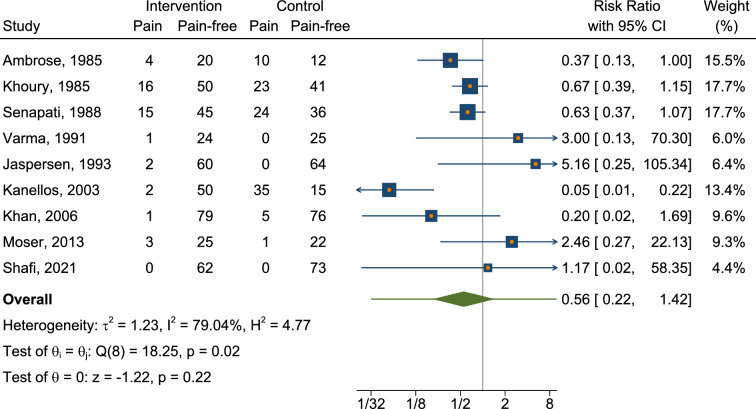
Forest plot showing rates of pain

**Fig. 5:**
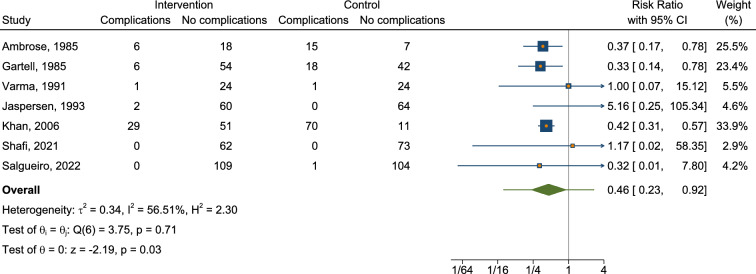
Forest plot showing rates of complications (overall)

**Fig. 6:**
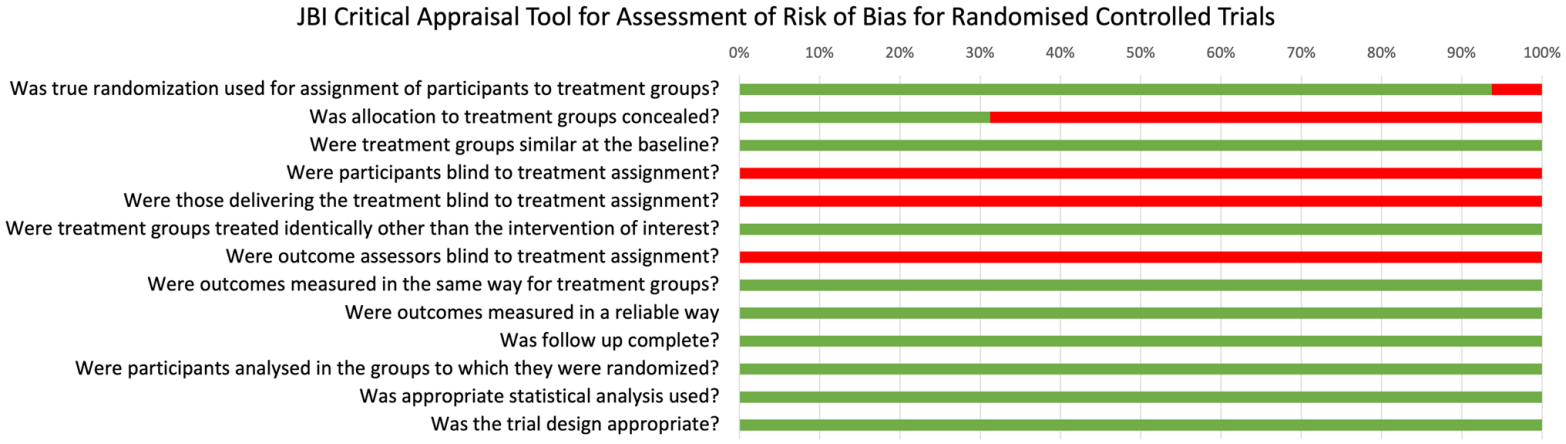
JBI Critical Appraisal Tool for Assessment of Risk of Bias for Randomized Controlled Trials

We conducted a meta-analysis of available RCTs to provide a quantitative assessment of sclerotherapy outcomes. Fourteen RCTs were identified that assessed the outcomes of sclerotherapy administered through the anoscope (Table 2). Notably, two additional RCTs describing sclerotherapy administered endoscopically were excluded from the quantitative meta-analysis because of methodological differences (i.e., study population consisting of patients with liver cirrhosis [29] or injection of an uncommon product [31]). One further RCT was excluded because of unconventional patients’ position (lithotomy rather than left lateral) [34]. The control treatments in the RCT included various interventions such as photocoagulation, rubber band ligation (RBL), bulking laxative, electrocoagulation, venotonic flavonoid, polidocanol liquid, alkaline of* Achyranthes aspera* Linn., 5% phenol almond oil, and HAL-RAR under local anesthesia, with some treatments administered in multiple sessions and under Doppler guidance. In terms of success rates, our meta-analysis revealed that sclerotherapy was not inferior to control interventions, with a risk ratio of 1.00 (95% CI 0.71–1.41) (Fig. 2, Appendix 1). The analysis showed substantial heterogeneity (*I*^2^ = 97.44%), and the test of group differences indicated no statistically significant variation (*Q*_b_(2) = 0.18, *p* = 0.91). We also analyzed the recurrence rate based on data from a total of four available studies (Fig. 3, Appendix 2). The risk ratio was 1.11 (95% CI 0.69–1.77), with moderate heterogeneity (*I*^2^ = 51.82%). A pooled risk ratio of 0.56 was estimated for the association between pain and intervention, but the evidence of less pain in the sclerotherapy group compared to control was not statistically significant (95% CI 0.22–1.42) based on data from nine available studies (Fig. 4, Appendix 3). The analysis revealed substantial heterogeneity (*I*^2^ = 79.04%). Our meta-analysis showed a significant reduction in overall complications following sclerotherapy compared to control interventions, with a risk ratio of 0.46 (95% CI 0.23–0.92) based on data from seven available studies (Fig. 5, Appendix 4). The analysis indicated moderate heterogeneity (*I*^2^ = 56.51%). Upon review, the primary flaw identified across the included studies predominantly pertained to blinding issues. Both participants and operators involved in performing the procedures or assessing outcomes often lacked adequate blinding, which could potentially introduce bias into the results (Fig. 6).

**Table 2:** Randomized controlled trials assessing the anoscopic injection of sclerotherapy

First author, reference	Type of treatments	
	Index	Control
Ambrose [20]	5% phenol	Photocoagulation
Khoury [21]	5% phenol (one session)	5% phenol (multiple sessions)
Gartell [22]	5% phenol	RBL
Senapati [23]	5% phenol	Bulking laxative
Varma [24]	5% phenol	Electrocoagulation
Jaspersen [25]	5% phenol	5% phenol under Doppler guidance
Kanellos [26]	5% phenol	RBL or sclerobanding
Khan [27]	5% phenol	Electrocoagulation
Yuksel [28]	3% polidocanol liquid	Venotonic flavonoid
Moser [14]	3% polidocanol foam	Polidocanol liquid
Shah [30]	3% polidocanol liquid	Alkaline of *Achyranthes aspera* Linn.
Shafi [32]	3% polidocanol liquid	5% phenol almond oil
Neves [33]	3% polidocanol foam	HAL-RAR under local anesthesia
Salgueiro [3]	3% polidocanol foam	RBL

*RBL* rubber band ligation, *HAL-RAR* hemorrhoidal artery ligation and recto anal repair

3 RCTs were not included in the quantitative meta-analysis because of different patient populations (patients with coexistent liver cirrhosis [29] or injection of an uncommon product [31]) or unconventional patients’ position (lithotomy rather than left lateral) [34]

## Discussion

In this systematic review, we examined the safety and efficacy of various sclerotherapy methods for HD over the past four decades. We conducted a comprehensive analysis of 44 selected studies encompassing 9729 patients, including 17 RCTs. The data analysis revealed that sclerotherapy administered through anoscopy was compared against various control interventions, including photocoagulation, RBL, bulking laxative, electrocoagulation, venotonic flavonoid, polidocanol liquid, alkaline of* Achyranthes aspera* Linn., 5% phenol almond oil, and HAL-RAR under local anesthesia in RCTs. The meta-analysis demonstrated that sclerotherapy yielded comparable success rates to these control interventions, with trends toward reduced pain and overall complications, despite issues related to blinding in the included studies.

It is noteworthy that a temporal trend has emerged in recent years, with a notable shift towards the use of polidocanol over 5% phenol oil. This shift is particularly evident when comparing studies from the last two decades to earlier research. From 2006 onwards, only one study has described the use of 5% phenol oil, in contrast to a more frequent mention of this agent in older studies dating back to 1985. This temporal evolution suggests a growing preference for polidocanol as a sclerosing agent in the treatment of HD, possibly due to its perceived increased safety.

The use of polidocanol in foam form offers several advantages over liquid agents. The foam formulation allows for a reduced injected dose of the sclerosing agent as a result of its larger volume, which increases the area of contact with the endothelium [16, 47]. This may contribute to potentially improved sclerotherapy outcomes and enhanced patient satisfaction. Our results align with the increasing evidence supporting the use of polidocanol foam, which has become a frequently utilized agent in recent RCTs [3, 33].

Our study also revealed that most of the included studies were conducted in Japan, suggesting a preference for sclerotherapy in this region [8, 35–42]. This observation may be attributed to cultural factors, variations in healthcare practices, or the availability of specific sclerosing agents. Further research from diverse geographical regions is needed to evaluate the generalizability of our findings and to explore potential regional variations in sclerotherapy practices.

It is worth noting that the variable “procedure duration” has only recently started to be consistently recorded in studies, reflecting an evolving trend in research methodology. Similarly, the utilization of validated scoring systems specific to HD has been a relatively recent development, with only seven studies incorporating these systems in the last 2 years. These advancements in data collection and standardized assessment tools are promising steps towards enhancing the comprehensiveness and comparability of future research in this field.

The comprehensive analysis of procedure-related aspects in this review, such as the route of administration, injected agent, needle caliber, site of injection, and patient positioning, underscores the need for a consensus to standardize clinical practice and reduce heterogeneity in sclerotherapy techniques.

Pain and bleeding were the most commonly reported perioperative issues, with pain occurring in approximately 8% of cases [14, 27, 48, 59]. These complications were generally mild and manageable. The overall satisfaction rates among patients undergoing sclerotherapy were high, emphasizing the positive impact of this procedure on patient outcomes. Long-term follow-up is necessary to assess the durability of treatment outcomes and to determine the recurrence rates associated with different sclerotherapy methods.

It is important to acknowledge the limitations of our study. The majority of included studies were retrospective or non-randomized, which may introduce selection bias and confounding factors. Additionally, the heterogeneity among the studies regarding patient characteristics, treatment protocols, and outcome measures limits the ability to perform a quantitative meta-analysis for all outcomes of interest. Heterogeneity exists even among the studies specifically focused on polidocanol foam regarding the various methods of foam preparation, including but not limited to the Tessari technique, the Easy Foam Kit, and Varixio. Standardization of foam preparation protocols in future studies could help reduce this source of heterogeneity and provide more consistent evidence on the effectiveness of polidocanol foam sclerotherapy. In addition to the aforementioned limitations, it is noteworthy that in our meta-analysis of the 14 RCTs, we had to group various “control treatments” under a single term, even though these control groups represented different conservative and non-excisional approaches to HD management. While all control treatments were non-surgical and aimed at conservative management of HD, the specific interventions within these groups varied. This grouping was required to allow analysis of the available data for analysis and should be taken into account when interpreting the results of our meta-analysis. Furthermore, we recognize the importance of considering individual patient circumstances and preferences, as supported by existing guidelines [60, 61]. Future well-designed studies are needed to address these limitations and provide a more comprehensive understanding of the role of sclerotherapy in the management of HD. Future well-designed RCTs are needed to provide more robust evidence on the safety and efficacy of sclerotherapy techniques.

## Conclusion

This systematic review and meta-analysis contributes valuable insights into the safety and efficacy of sclerotherapy for HD, highlighting its comparable success rates, potential for reduced pain, and lower overall complication rates when compared to control interventions.

These findings support the consideration of sclerotherapy as a valid alternative to conventional surgical interventions, particularly in patients with lower-grade hemorrhoids or those who are not suitable candidates for surgery. Further research is warranted to optimize sclerotherapy techniques, standardize protocols, and evaluate long-term outcomes.

## Supplementary Information

Below is the link to the electronic supplementary material.

Appendix 1 Funnel plot showing rates of success. Supplementary file1 (PDF 55 KB)

Appendix 2 Funnel plot showing rates of recurrence. Supplementary file2 (PDF 54 KB)

Appendix 3 Funnel plot showing rates of pain. Supplementary file3 (PDF 54 KB)

Appendix 4 Funnel plot showing rates of complications (overall). Supplementary file4 (PDF 54 KB)

## Data Availability

The data that support the findings of this study are available from the corresponding authors upon reasonable request.
