# Supplementary figures and images for: Sclerotherapy for hemorrhoidal disease: systematic review and meta-analysis
Source: Tech Coloproctol. 2024 Jan 23;28(1):28. doi: 10.1007/s10151-023-02908-w (PMC10806988; doi:10.1007/s10151-023-02908-w)

# Contour-enhanced funnel plot

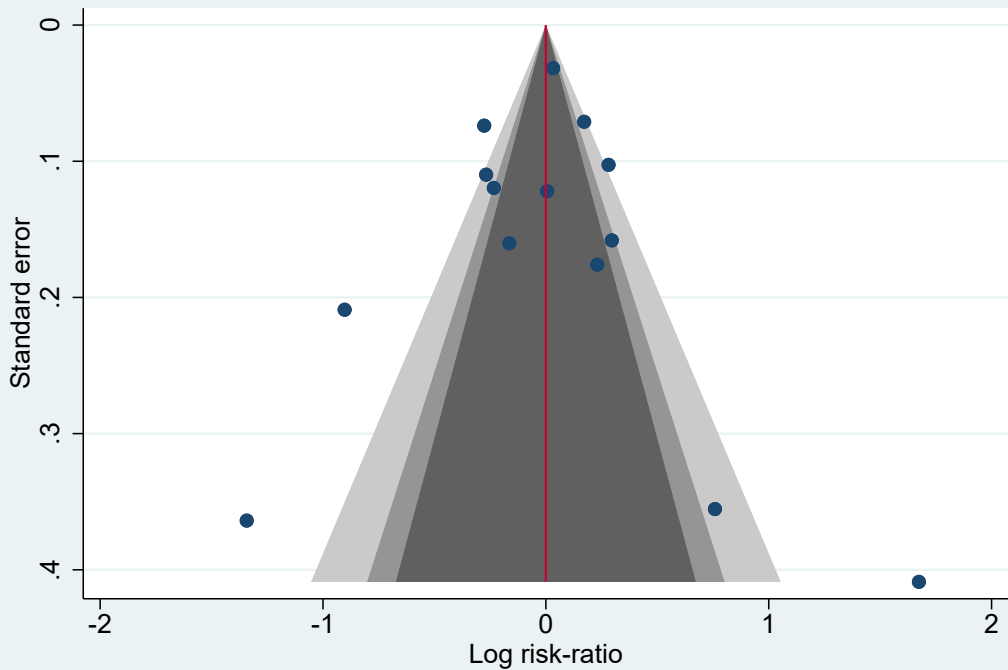

Supplement: Supplementary file 1 — Appendix 1 Funnel plot showing rates of success. Supplementary file1 (PDF 55 KB) [file 10151_2023_2908_MOESM1_ESM.pdf]

# Contour-enhanced funnel plot

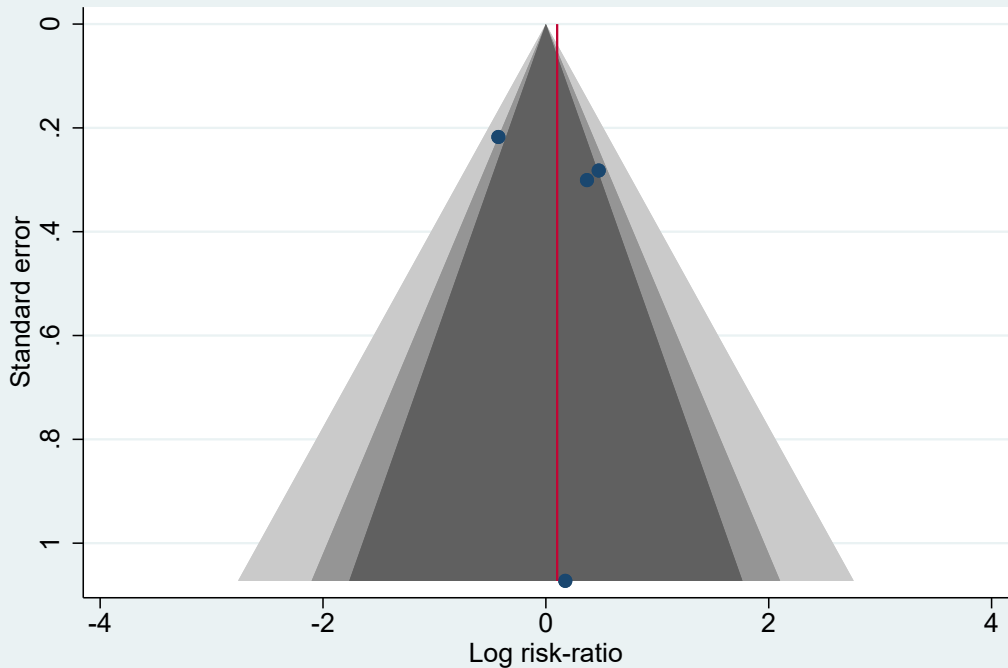

Supplement: Supplementary file 2 — Appendix 2 Funnel plot showing rates of recurrence. Supplementary file2 (PDF 54 KB) [file 10151_2023_2908_MOESM2_ESM.pdf]

# Contour-enhanced funnel plot

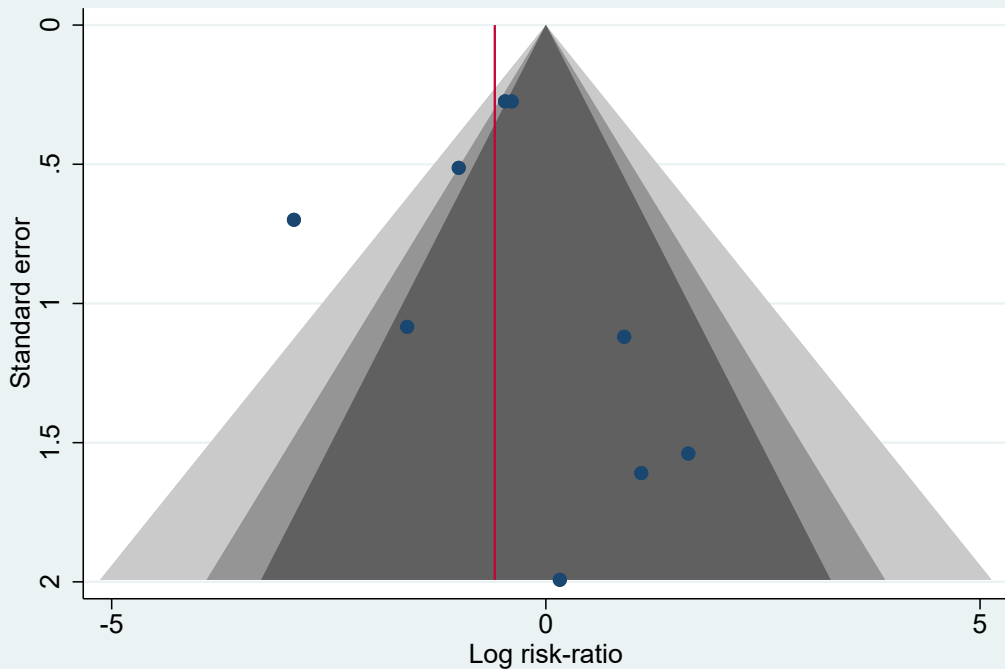

Supplement: Supplementary file 3 — Appendix 3 Funnel plot showing rates of pain. Supplementary file3 (PDF 54 KB) [file 10151_2023_2908_MOESM3_ESM.pdf]

# Contour-enhanced funnel plot

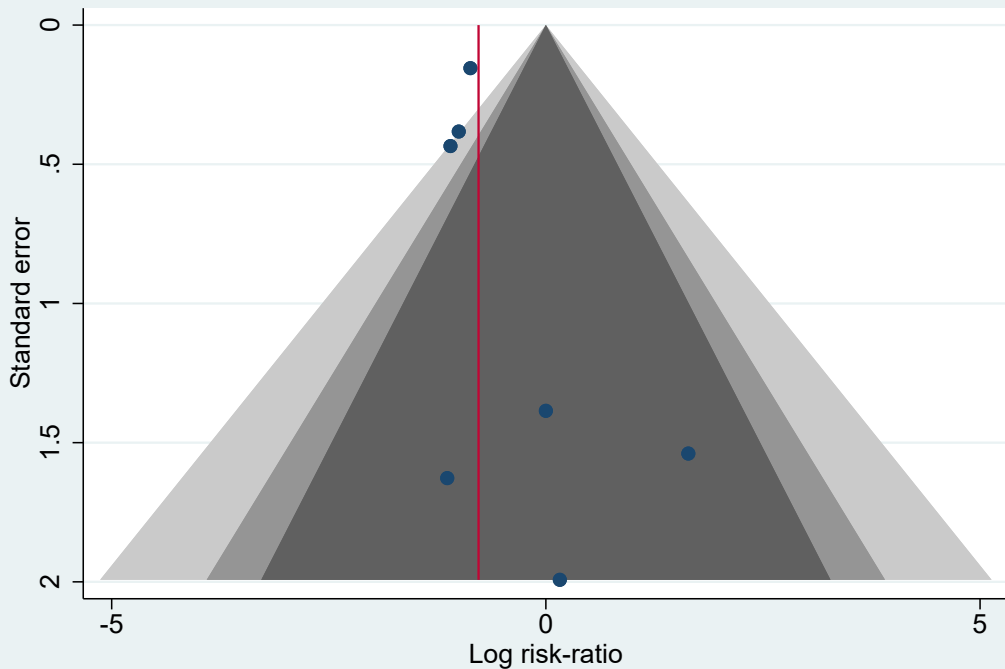

Supplement: Supplementary file 4 — Appendix 4 Funnel plot showing rates of complications (overall). Supplementary file4 (PDF 54 KB) [file 10151_2023_2908_MOESM4_ESM.pdf]
